# Identifying important motivational factors for professionals in Greek hospitals

**DOI:** 10.1186/1472-6963-9-164

**Published:** 2009-09-15

**Authors:** Nick Kontodimopoulos, Victoria Paleologou, Dimitris Niakas

**Affiliations:** 1 Faculty of Social Sciences, Hellenic Open University, Bouboulinas 57, 26222, Patras, Greece

## Abstract

**Background:**

The purpose of this study was to identify important motivational factors according to the views of health-care professionals in Greek hospitals and particularly to determine if these might differ in the public and private sectors.

**Methods:**

A previously developed -and validated- instrument addressing four work-related motivators (job attributes, remuneration, co-workers and achievements) was used. Three categories of health care professionals, doctors (N = 354), nurses (N = 581) and office workers (N = 418), working in public and private hospitals, participated and motivation was compared across socio-demographic and occupational variables.

**Results:**

The range of reported motivational factors was mixed and Maslow's conclusions that lower level motivational factors must be met before ascending to the next level were not confirmed. The highest ranked motivator for the entire sample, and by professional subgroup, was achievements (*P *< 0.001). Within subgroups, motivators were similar, and only one significant difference was observed, namely between doctors and nurses in respect to co-workers (*P *< 0.05). Remuneration (and salary in particular) was reported as a significant incentive only for professionals in managerial positions. Health professionals in private hospitals were motivated by all factors significantly more than their public-hospital counterparts.

**Conclusion:**

The results are in agreement with the literature which focuses attention to management approaches employing both monetary and non-monetary incentives to motivate health care workers. This study showed that intrinsic factors are particularly important and should become a target for effective employee motivation.

## Background

The first evidence that employees are not motivated solely by money and that employee behavior is linked to attitudes was a result of research (referred to as *the Hawthorne Studies*) conducted by Elton Mayo from 1924 to 1932 [1]. These studies constituted the initiation of the human relations approach to management, whereby employees' needs and motivation become managers' primary focus [2]. In the following decades, many researchers addressed the issue of what motivated employees and how they were motivated. Five major approaches having led to a better understanding of motivation are Maslow's need-hierarchy theory [3], Herzberg's two-factor theory [4], Vroom's expectancy theory [5], Adams' equity theory [6], and Skinner's reinforcement theory [7].

Contemporary studies have given various definitions to motivation, such as a psychological process that gives behavior purpose and direction [8], an internal drive to satisfy an unsatisfied need [9], and the will to achieve. Motivation is an internal driving force that is not easily influenced by external factors. However, managers can satisfy employees so they become motivated and of all the functions a manager performs, motivating employees is arguably the most complex, since it is influenced by both financial and non-financial incentives [10] due, in part, to the fact that what motivates employees changes constantly [11]. The terms *job satisfaction *and *motivation *are often used interchangeably, however there is a borderline. Job satisfaction is a person's emotional response to his or her job condition, whereas motivation is the driving force to pursue and satisfy needs. The need for motivation stems from the need for survival and motivated employees help organizations survive [12].

In Greece, human resources management practices in the public sector are centralized to a great extent. Recruitment and selection is conducted by a supreme council for personnel selection, and both rewards and promotions are linked to demographic characteristics and controlled by central authorities. Consequently, the top management is practically unable to ensure employee motivation, reward or punishment. This problem is supplemented by a lack of internal operation procedures, causing ineffective communication and confusing tasks [13]. Organizational culture in Greek public hospitals is not very strong, perhaps because managers cannot infuse a common pattern of values among employees, as they are practically unable to form human resource policies and practices [14]. Furthermore, a significant number of current hospital employees were recruited under previous bureaucratic order, and their values, expectations and standards are still influenced by previous policies and practices [15].

The situation is different in the private hospitals, where management is free to hire employees according to their own criteria, which typically revolve around profit-making for the organization. The main sources of income in the private sector are payments from private insurance and out-of-pocket payments by the patients. Health care professionals, especially the physicians, are recruited on the basis of their commitment to increasing hospital revenue and can be motivated accordingly to adhere to this goal by appropriate human resource management policies which are set by hospital management. Appropriate motivation policies can be set for nurses and office workers as well.

The purpose of this study was to identify important motivational factors according to the views of health-care professionals in Greek hospitals and to determine how these might differ according to socio-demographic and job-related factors. A previously developed -and validated- instrument [16] addressing four work-related motivators (job attributes, remuneration, co-workers and achievements) was used. Three categories of health care professionals, i.e. doctors, nurses and office workers, working in public and private hospitals, participated and motivation (expressed numerically) was compared across socio-demographic and occupational variables. The main focus was on potential motivational differences between professionals in the public and private sectors. The results are expected to have significant policymaking implications for hospital administrations

## Methods

### Instrument

The processes leading to the development of the instrument used in the present study have been described in detail elsewhere [16]. In brief, a Medline search returned studies on multidimensional job satisfaction and/or motivation instruments. Based on the reported results, the instruments were assessed on the basis of reliability, content/construct validity and responsiveness and were the basis for constructing the present instrument. Item selection was guided by Maslow and Herzberg's theories and the initial version of the instrument contained 48 items. To enhance content validity, three experts in human resource management and psychometry reviewed the items for appropriateness, clarity and completeness, and the instrument in its entirety for appearance, item sequence and completion time. The procedure was repeated with three other experts resulting in the removal of 20 vague, ambiguous, redundant and irrelevant items. The resulting 28-item instrument was pilot-tested in an Athens general hospital, using a random sample of 74 physicians, nurses and hospital administrative staff, and instrument straightforwardness, brevity and acceptance were confirmed.

The 28-item version was subjected to factor analysis which identified the underlying constructs, and tests of scaling assumptions, according to the Multitrait-Multimethod Matrix, were used to confirm the hypothesized component structure. Four components -labeled *job attributes*, *remuneration*, *co-workers *and *achievement-*, referring to intrinsic individual needs and external job-related aspects, explained 59.61% of the variability. Scale reliability ranged from 0.782 to 0.901 and internal item consistency and discriminant validity criteria were satisfied. Nine items not meeting item-scale criteria were removed, resulting in the 19-item instrument used in the present study.

The *job attributes *factor addresses 7 items: authority, goals, creativity opportunities, clear duties, job control, skill exploitation and decision-making. The *remuneration *factor addresses 4 items: salary, environment, retirement/pension and absenteeism. The *co-workers *factor addresses 5 items: teamwork, job pride, appreciation, supervisor and fairness. Finally, the *achievements *factor addresses 3 items: job meaningfulness, earned respect and interpersonal relationships [16]. All items were neutrally phrased as "*In your case, how important is........ for increasing your will to perform better at work?"*. Responses were provided on a five-point unipolar adjective scale, with 1 corresponding to *"not at all"*, 2 to *"a little bit"*, 3 to *"moderately"*, 4 to *"very" *and 5 to *"extremely"*. The survey also included questions on age, gender, education, position, years of experience and department.

### Sample and data collection

The aim was to obtain a representative sample of the Greek health care workers from public and private hospitals. In the public sector the target was 10% of NHS facilities (approximately 130), while keeping in mind the geographical distribution of hospitals. Hence, 13 hospitals were randomly selected and approached for participation in the study. Eleven hospital administrations agreed, whereas two declined on the basis that the survey could disrupt the workers' concentration. Two alternative hospitals, similar in characteristics to the ones declining, were randomly chosen and agreed to permit the survey. Therefore, 13 public (NHS) hospitals were designated, 5 from the greater Athens area and 8 from various regions of the country, all of which were acute care facilities. Psychiatric and other specialty hospitals were excluded. As for the private sector, taking into consideration that these hospitals in Greece are overwhelmingly located in Athens, we chose (once again randomly) five large facilities, out of which four agreed to participate.

In each of the seventeen hospitals, the survey was approved by the Review Boards and permission to administer the survey to employees was granted by the hospitals' administrations. The study was conducted between February and June 2007, and 1600 questionnaires overall were distributed proportionally to facility size (number of beds) and staff synthesis. Overall, 450 doctors (physicians, residents and academics), 650 nurses (all education levels) and 500 office workers were selected, approximating the proportion of these subgroups in the Greek health system. The response rates were 78.7% for doctors (N = 354), 89.4% for nurses (N = 581) and 83.6% for office workers (N = 418). The absolute numbers of participants from each hospital ranged between 56 and 100. All participants (N = 1353) provided informed consent to participate and were self-administered the survey.

### Analysis

The sample was analyzed as a whole and by professional subgroup. For each motivation factor, summated scores were calculated on a 1-5 scale, with higher scores corresponding to a higher motivation to perform better. Nonparametric chi-square, Mann-Whitney and Kruskal-Wallis tests were used for comparisons according to gender, age, education, and job-related variables such as hospital ownership (i.e. public or private), years spent in the hospital and in the current position and managerial position. Multivariate analyses with each motivation factor the dependent variable, and sociodemographic and work-related variables as independent predictors were conducted. All analyses were performed with SPSS, version 15.0 (SPSS Inc., Chicago IL).

## Results

Sample distribution, according to demographic and work-related variables, is shown in Table 1. The majority of respondents were female (69.4%), mostly due to the large number of female nurses and the mean age for the whole sample was 39.9 years. The mean time spent in the particular hospital was 11.7 years and in the current position 7.9 years. Overall, 76.6% of the respondents worked in public hospitals and 22.6% were responsible for managing other people. By subgroup, doctors were dominantly males (65.9%), whereas office workers and especially nurses were females (71.6% and 89.1% respectively). The mean age of doctors, nurses and office workers was 44.6 (± 9.2), 36.6 (± 8.3) and 40.5 (± 8.2) respectively. Most of the doctors had been in the present hospital for ≤ 5 years and the office workers for >15 years (chi-square, *P *< 0.001). In all three professional groups, the majority of respondents were in the same working position for ≤ 5 years (chi-square, *P *< 0.001).

**Table 1:** Variation of demographic and job-related characteristics by category of health worker

**Demographic and job-related variables**	**Frequency (% valid)**			
	
	**Overall** **(N = 1353)**	**Doctors** **(N = 354)**	**Nurses** **(N = 581)**	**Office workers** **(N = 418)**
	
Gender				
Male	413 (30.6)	232 (65.9)	63 (10.9)	118 (28.4)
Female	935 (69.4)	120 (34.1)	517 (89.1)	298 (71.6)
				
Age				
≤ 35	502 (37.1)	94 (26.6)	271 (46.6)	137 (32.8)
36-49	648 (47.9)	149 (42.1)	268 (46.1)	231 (55.2)
≥ 50	203 (15.0)	111 (31.3)	42 (7.2)	50 (12.0)
				
Education				
High school	379 (28.1)	---	156 (26.9)	223 (53.5)
University	796 (59.0)	221 (62.6)	409 (70.7)	166 (39.8)
Postgraduate	174 (12.9)	134 (37.4)	14 (2.4)	28 (6.7)
				
Years in workplace				
≤ 5 years	506 (37.4)	164 (46.3)	206 (35.5)	136 (32.5)
6-15 years	365 (27.0)	96 (27.1)	178 (30.6)	91 (21.8)
> 15 years	482 (35.6)	94 (26.6)	197 (33.9)	191 (45.7)
				
Years in same position				
≤ 5 years	618 (45.7)	164 (46.3)	251 (43.2)	203 (48.6)
6-15 years	431 (31.8)	114 (32.2)	189 (32.5)	128 (30.6)
> 15 years	304 (22.5)	76 (21.5)	141 (24.3)	87 (20.8)
				
Hospital status				
Public	1037 (76.6)	284 (80.2)	425 (73.1)	328 (78.5)
Private	316 (23.4)	70 (19.8)	156 (26.9)	90 (21.5)
				
Management position				
Yes	306 (22.6)	102 (28.9)	135 (23.3)	69 (16.5)
No	1045 (77.4)	251 (71.1)	445 (76.7)	349 (83.5)

The mean scores for each motivating factor are shown in Table 2. The number one ranked motivator was achievements which was significantly higher than all the others both for the overall sample, and by professional subgroup (Wilcoxon test, *P *< 0.001). The next ranked motivator was remuneration, except for nurses which ranked co-workers as the second strongest motivating factor. However, the score differences for these two factors were insignificant in all groups. The least ranked motivator by all subgroups was job attributes for which the mean score was significantly lower (Wilcoxon test, *P *< 0.001) than all the others. Within the subgroups, the scores for each motivator were astonishingly similar, and only one significant difference was observed, namely between doctors and nurses in respect to the "co-workers" scores (Wilcoxon test, *P *< 0.05).

**Table 2:** Mean scores^1 ^(SD) by motivating factor for the entire sample and by category of health worker

	**Overall** **(N = 1353)**	**Doctors** **(N = 354)**	**Nurses** **(N = 581)**	**Office workers** **(N = 418)**
	
Job Attributes	3.37 (1.01)	3.45 (0.93)	3.38 (1.02)	3.28 (1.03)
Remuneration	3.72 (1.06)	3.66 (1.03)	3.72 (1.09)	3.77 (1.04)
Co-workers	3.71 (0.88)	3.62 (0.89)	3.76 (0.86)	3.73 (0.90)
Achievements	4.28 (0.84)	4.28 (0.79)	4.27 (0.86)	4.28 (0.84)

^1 ^Reported on a 1-5 scale with higher values corresponding to higher motivation

Job attributes addresses intrinsic motivators such as decision-making, creativity and skill exploitation, i.e. internal self-needs which must be satisfied before experiencing true job satisfaction. This factor, according to respondents in managerial positions, was a significant motivator (Table 3) (Kruskal-Wallis, *P *< 0.01). Hospital ownership significantly differentiated the motivational effect of job attributes for office workers (Mann-Whitney, *P *< 0.05). Physicians working for >15 years in the same hospital were more motivated by this factor (Kruskal-Wallis, *P *< 0.05), compared to colleagues with less years. As for the demographics, only education had a significant influence on motivation of office workers, especially those with postgraduate degrees (Kruskal-Wallis, *P *< 0.05).

**Table 3:** Mean scores^1 ^(SD) by demographic and job-related characteristics for the "job attributes" motivator

	**Motivating Factor: JOB ATTRIBUTES**			
	
**Demographic variables**	**Overall** **(N = 1353)**	**Doctors** **(N = 354)**	**Nurses** **(N = 581)**	**Office workers** **(N = 418)**
	
Gender				
Male	3.42 (0.97)	3.48 (0.95)	3.37 (1.01)	3.33 (0.99)
Female	3.35 (1.02)	3.42 (0.92)	3.38 (1.03)	3.26 (1.05)
*P-sig*^2^.	*0.372*	*0.555*	*0.851*	*0.730*
Age				
≤ 35	3.36 (0.97)	3.25 (1.05)	3.40 (0.97)	3.36 (0.98)
36-49	3.33 (1.04)	3.47 (0.92)	3.36 (1.07)	3.20 (1.06)
≥ 50	3.52 (0.94)	3.61 (0.83)	3.39 (1.05)	3.46 (1.05)
*P*-*sig*^3^.	*0.081*	*0.083*	*0.912*	*0.179*
Education				
High school	3.22 (1.05)	---	3.28 (1.03)	3.18 (1.07)
University	3.40 (0.99)	3.42 (0.94)	3.40 (1.02)	3.38 (0.96)
Postgraduate	3.56 (0.94)	3.52 (0.92)	3.88 (0.88)	3.57 (1.05)
*P*-*sig*^3^.	*0.001*	*0.431*	*0.088*	*0.042*
Job-related variables				
				
Years in workplace				
≤ 5 years	3.35 (0.96)	3.32 (0.97)	3.31 (0.96)	3.46 (0.94)
6-15 years	3.33 (1.04)	3.47 (0.92)	3.36 (1.07)	3.12 (1.07)
> 15 years	3.42 (1.03)	3.66 (0.85)	3.47 (1.04)	3.24 (1.07)
*P*-*sig*^3^.	*0.035*	*0.032*	*0.204*	*0.083*
Years in same position				
≤ 5 years	3.37 (0.99)	3.38 (0.94)	3.40 (1.00)	3.31 (1.04)
6-15 years	3.34 (1.02)	3.54 (0.89)	3.30 (1.05)	3.22 (1.06)
> 15 years	3.42 (1.01)	3.47 (0.99)	3.46 (1.03)	3.31 (0.99)
*P*-*sig*^3^.	*0.552*	*0.468*	*0.375*	*0.775*
Hospital status				
Public	3.32 (1.04)	3.41 (0.93)	3.35 (1.07)	3.21 (1.07)
Private	3.52 (0.88)	3.63 (0.93)	3.46 (0.88)	3.56 (0.83)
*P-sig*^2^.	*0.006*	*0.058*	*0.429*	*0.011*
Management position				
Yes	3.69 (0.87)	3.68 (0.93)	3.65 (0.92)	3.79 (0.68)
No	3.28 (1.02)	3.36 (0.92)	3.30 (1.04)	3.18 (1.06)
*P-sig*^2^.	*<0.001*	*0.002*	*0.001*	*<0.001*

^1 ^Reported on a 1-5 scale with higher values corresponding to higher motivation

^2^According to Mann-Whitney test

^3^According to Kruskal-Wallis test

Scores by demographic and work-related variables for the remuneration factor, which addresses extrinsic motivators such as salary, benefits, pension, insurance, etc., are shown in Table 4. Interestingly, the motivating effect of remuneration was significantly different only by hospital ownership, and appeared not to be influenced by other sociodemographic or work-related variables. Specifically, all personnel categories working in private hospitals were much more motivated by a good remuneration package compared to their public hospital counterparts, with statistically significant differences observed in the case of physicians (*P *< 0.01) and nurses (*P *< 0.05), and a borderline significant difference for the office workers (*P *= 0.080).

**Table 4:** Mean scores^1 ^(SD) by demographic and job-related characteristics for the "remuneration" motivator

**Demographic variables**	**Motivating Factor: REMUNERATION**			
	
	**Overall** **(N = 1353)**	**Doctors** **(N = 354)**	**Nurses** **(N = 581)**	**Office workers** **(N = 418)**
	
Gender				
Male	3.76 (0.98)	3.73 (0.99)	3.93 (0.91)	3.75 (0.99)
Female	3.70 (1.09)	3.56 (1.09)	3.69 (1.11)	3.77 (1.06)
*P-sig*^2^.	*0.629*	*0.219*	*0.248*	*0.660*
Age				
≤ 35	3.72 (1.01)	3.57 (1.20)	3.70 (1.02)	3.87 (0.83)
36-49	3.74 (1.09)	3.78 (0.95)	3.76 (1.17)	3.70 (1.09)
≥ 50	3.63 (1.08)	3.58 (0.96)	3.56 (1.11)	3.81 (1.29)
*P*-*sig*^3^.	*0.342*	*0.247*	*0.230*	*0.388*
Education				
High school	3.77 (1.08)	---	3.88 (1.00)	3.69 (1.13)
University	3.70 (1.07)	3.63 (1.07)	3.65 (1.12)	3.91 (0.88)
Postgraduate	3.70 (0.98)	3.73 (0.94)	3.71 (1.05)	3.61 (1.11)
*P*-*sig*^3^.	*0.426*	*0.589*	*0.138*	*0.275*
Job-related variables				
				
Years in workplace				
≤ 5 years	3.76 (1.00)	3.70 (1.10)	3.66 (0.99)	3.96 (0.87)
6-15 years	3.70 (1.05)	3.60 (1.03)	3.72 (1.10)	3.78 (0.94)
> 15 years	3.69 (1.13)	3.66 (0.88)	3.77 (1.19)	3.62 (1.17)
*P*-*sig*^3^.	*0.848*	*0.476*	*0.187*	*0.078*
Years in same position				
≤ 5 years	3.76 (1.02)	3.73 (1.04)	3.71 (1.07)	3.84 (0.95)
6-15 years	3.75 (1.04)	3.67 (0.98)	3.80 (1.04)	3.74 (1.09)
> 15 years	3.60 (1.16)	3.52 (1.05)	3.61 (1.20)	3.65 (1.17)
*P*-*sig*^3^.	*0.266*	*0.277*	*0.541*	*0.713*
Hospital status				
Public	3.64 (1.11)	3.59 (1.06)	3.63 (1.16)	3.70 (1.10)
Private	3.98 (0.82)	3.97 (0.81)	3.97 (0.86)	4.01 (0.74)
*P-sig*^2^.	*<0.001*	*0.008*	*0.011*	*0.080*
Management position				
Yes	3.70 (1.06)	3.69 (1.05)	3.60 (1.13)	3.89 (0.92)
No	3.72 (1.06)	3.65 (1.02)	3.75 (1.08)	3.74 (1.06)
*P-sig*^2^.	*0.681*	*0.634*	*0.222*	*0.417*

^1 ^Reported on a 1-5 scale with higher values corresponding to higher motivation

^2^According to Mann-Whitney test

^3^According to Kruskal-Wallis test

The co-workers motivator (Table 5) refers to working relationships with supervisors and colleagues as a source of satisfaction and motivation. For doctors, increasing age was linked to valuing good working relationships higher, although the effect was just borderline significant (*P *= 0.058). Years spent in the same hospital significantly differentiated the motivational effect of this factor for doctors (*P *< 0.05), in favor of those working >15 years. Satisfaction and motivational drive also stemmed from good interpersonal and working relationships for the office workers in the same position for >15 years (*P *< 0.05). The latter (*P *< 0.01) and nurses as well (*P *< 0.05), were also affected by hospital ownership in terms of motivation by the co-workers factor and, for both, motivation was higher in the private hospitals. In terms of the entire sample, good professional relationships motivated professionals in managerial positions (*P *< 0.001).

**Table 5:** Mean scores^1 ^(SD) by demographic and job-related characteristics for the "co-workers" motivator

	**Motivating Factor: CO-WORKERS**			
	
**Demographic variables**	**Overall** **(N = 1353)**	**Doctors** **(N = 354)**	**Nurses** **(N = 581)**	**Office workers** **(N = 418)**
	
Gender				
Male	3.72 (0.89)	3.68 (0.89)	3.89 (0.82)	3.73 (0.91)
Female	3.71 (0.88)	3.52 (0.88)	3.74 (0.87)	3.72 (0.90)
*P-sig*^2^.	*0.767*	*0.085*	*0.256*	*0.938*
Age				
≤ 35	3.71 (0.87)	3.49 (0.98)	3.77 (0.84)	3.75 (0.81)
36-49	3.69 (0.91)	3.58 (0.86)	3.75 (0.90)	3.68 (0.95)
≥ 50	3.81 (0.85)	3.80 (0.83)	3.76 (0.80)	3.88 (0.94)
*P*-*sig*^3^.	*0.276*	*0.058*	*0.940*	*0.256*
Education				
High school	3.77 (0.93)	---	3.79 (0.87)	3.75 (0.97)
University	3.71 (0.85)	3.63 (0.88)	3.75 (0.85)	3.71 (0.82)
Postgraduate	3.62 (0.93)	3.62 (0.92)	3.63 (1.11)	3.61 (0.91)
*P*-*sig*^3^.	*0.110*	*0.985*	*0.794*	*0.526*
Job-related variables				
	
Years in workplace				
≤ 5 years	3.70 (0.85)	3.53 (0.92)	3.73 (0.84)	3.87 (0.73)
6-15 years	3.70 (0.91)	3.57 (0.90)	3.76 (0.86)	3.72 (1.00)
> 15 years	3.73 (0.90)	3.85 (0.80)	3.78 (0.88)	3.63 (0.96)
*P*-*sig*^3^.	*0.761*	*0.023*	*0.815*	*0.144*
Years in same position				
≤ 5 years	3.67 (0.88)	3.61 (0.91)	3.73 (0.88)	3.65 (0.86)
6-15 years	3.70 (0.91)	3.63 (0.87)	3.73 (0.83)	3.73 (1.04)
> 15 years	3.82 (0.85)	3.65 (0.89)	3.84 (0.87)	3.91 (0.77)
*P*-*sig*^3^.	*0.085*	*0.965*	*0.411*	*0.046*
Hospital status				
Public	3.66 (0.91)	3.60 (0.90)	3.70 (0.86)	3.67 (0.95)
Private	3.88 (0.78)	3.74 (0.87)	3.92 (0.78)	3.94 (0.69)
*P-sig*^2^.	*<0.001*	*0.168*	*0.009*	*0.028*
Management position				
Yes	3.82 (0.85)	3.75 (0.89)	3.81 (0.87)	3.92 (0.74)
No	3.69 (0.89)	3.58 (0.88)	3.74 (0.86)	3.69 (0.93)
*P-sig*^2^.	*0.029*	*0.081*	*0.432*	*0.091*

^1 ^Reported on a 1-5 scale with higher values corresponding to higher motivation

^2^According to Mann-Whitney test

^3^According to Kruskal-Wallis test

Scores for the achievements factor, which refers to intrinsic motivators such as pride, appreciation, respect and social acceptance, are shown in Table 6. The most important determining variable was age, which was positively associated with higher motivation for the entire sample (*P *< 0.001), doctors (*P *< 0.05) and nurses (*P *< 0.01). More years in the same hospital and in the same position meant higher motivation by achievements for the overall sample (*P *< 0.01 and *P *< 0.05 respectively) and for nurses specifically (*P *< 0.01 and *P *< 0.05 respectively). Physicians' motivation by achievements was affected by hospital ownership, in favor of private hospitals (*P *< 0.05). Finally, professional achievements highly motivated physicians and nurses in managerial positions (*P *< 0.01), and had a borderline significant effect on office workers as well (*P *= 0.068).

**Table 6:** Mean scores^1 ^(SD) by demographic and job-related characteristics for the "achievements" motivator

**Demographic variables**	**Motivating Factor: ACHIEVEMENTS**			
	
	Overall (N = 1353)	Doctors (N = 354)	Nurses (N = 581)	Office workers (N = 418)
	
Gender				
Male	4.29 (0.75)	4.30 (0.76)	4.33 (0.75)	4.27 (0.73)
Female	4.27 (0.88)	4.23 (0.86)	4.27 (0.87)	4.28 (0.87)
*P-sig*^2^.	*0.437*	*0.593*	*0.989*	*0.226*
Age				
≤ 35	4.18 (0.86)	4.06 (0.95)	4.17 (0.89)	4.29 (0.73)
36-49	4.31 (0.82)	4.29 (0.78)	4.36 (0.82)	4.28 (0.85)
≥ 50	4.40 (0.79)	4.45 (0.62)	4.40 (0.80)	4.29 (1.07)
*P*-*sig*^3^.	*<0.001*	*0.012*	*0.008*	*0.313*
Education				
High school	4.28 (0.89)	---	4.32 (0.77)	4.24 (0.97)
University	4.25 (0.83)	4.21 (0.85)	4.24 (0.89)	4.33 (0.64)
Postgraduate	4.39 (0.72)	4.38 (0.69)	4.55 (0.77)	4.35 (0.86)
*P*-*sig*^3^.	*0.102*	*0.120*	*0.183*	*0.480*
Job-related variables				
				
Years in workplace				
≤ 5 years	4.20 (0.84)	4.21 (0.84)	4.13 (0.92)	4.29 (0.73)
6-15 years	4.28 (0.83)	4.26 (0.89)	4.27 (0.86)	4.34 (0.81)
> 15 years	4.35 (0.83)	4.41 (0.71)	4.42 (0.77)	4.25 (0.93)
*P*-*sig*^3^.	*0.001*	*0.156*	*0.002*	*0.577*
Years in same position				
≤ 5 years	4.22 (0.86)	4.25 (0.80)	4.16 (0.93)	4.26 (0.81)
6-15 years	4.35 (0.76)	4.36 (0.69)	4.34 (0.76)	4.35 (0.84)
> 15 years	4.30 (0.88)	4.20 (0.91)	4.38 (0.82)	4.24 (0.94)
*P*-*sig*^3^.	*0.021*	*0.639*	*0.027*	*0.378*
Hospital status				
Public	4.25 (0.87)	4.23 (0.82)	4.25 (0.88)	4.25 (0.89)
Private	4.38 (0.72)	4.47 (0.66)	4.33 (0.80)	4.40 (0.62)
*P-sig*^2^.	*0.083*	*0.022*	*0.541*	*0.719*
Management position				
Yes	4.47 (0.70)	4.47 (0.70)	4.44 (0.76)	4.50 (0.57)
No	4.22 (0.87)	4.20 (0.82)	4.22 (0.88)	4.24 (0.88)
*P-sig*^2^.	*<0.001*	*0.001*	*0.008*	*0.068*

^1 ^Reported on a 1-5 scale with higher values corresponding to higher motivation

^2^According to Mann-Whitney test

^3^According to Kruskal-Wallis test

In a series of multivariate analyses, each motivational factor was regressed against socio-demographic (gender, age, education), and work related variables (years in hospital and current position, managing people, public/private hospital), and the results are presented in Table 7. Working in a private hospital was positively and significantly associated with higher scores in all motivational factors. This particular result was most evident for nurses (all factors except job attributes) and office workers (all factors except achievements). As for doctors, being employed in a private hospital resulted in higher scores in the remuneration and achievements motivational factors. The second important variable resulting from these analyses was managerial position, i.e. in all professional categories, those responsible for managing other people had significantly higher scores in the job attributes factor, as well as in the achievements factor (office workers only). Interestingly, achievements were the only motivational factor significantly affected by any other variables, specifically age and years in the workplace for doctors and nurses respectively.

**Table 7:** Multivariate analyses for motivation factors by professional category

	**B Coefficient *(p value)***											
	
	**JOB ATTRIBUTES**			**REMUNERATION**			**CO-WORKERS**			**ACHIEVEMENTS**		
				
**Model**	**Doctors**	**Nurses**	**Office Workers**	**Doctors**	**Nurses**	**Office Workers**	**Doctors**	**Nurses**	**Office Workers**	**Doctors**	**Nurses**	**Office Workers**
				
Constant	3.97 *(<0.001)*	3.93 *(<0.001)*	3.91 *(<0.001)*	3.29 *(<0.001)*	3.27 *(<0.001)*	3.33 *(<0.001)*	3.65 *(<0.001)*	3.46 *(<0.001)*	3.31 *(<0.001)*	3.25 *(<0.001)*	3.75 *(<0.001)*	4.73 *(<0.001)*
Female												
Age (per year)										0.016 *(0.001)*		
Education level												
Years in workplace											0.018 *(0.001)*	
Years in position												
Private hospital			0.407 *(0.001)*	0.331 *(0.016)*	0.357 *(0.001)*	0.353 *(0.006)*		0.219 *(0.012)*	0.324 *(0.004)*	0.257 *(0.017)*	0.239 *(0.013)*	
Managerial position	0.274 *(0.016)*	0.316 *(0.004)*	0.617 *(<0.001)*									0.249 *(0.032)*
R^2^=	0.016	0.015	0.072	0.016	0.019	0.018	0.009	0.010	0.019	0.042	0.019	0.010

## Discussion

Compared to other sectors of the world-wide economy, healthcare is undergoing a massive transformation due to a changing workforce, high cost and increased complexity of technology, increased demand from the aging population, increased regulations, regulatory compliance and demand of continuous quality improvement, the consumer orientation of the industry, and various ongoing reorganizations [17]. It has been suggested that adapting a healthcare organization to these changes is the greatest challenge that administrators face and understanding the workers' needs becomes essential for promoting healthy working environments [18]. While resource availability and worker competencies are essential, they are not sufficient in themselves to ensure desired performance [19]. Worker motivation also depends upon the organizational context. Organizational structures, resources, processes, and culture, as well as organizational feedback about performance, contribute to motivational processes occurring at the individual level. The resourceful manager should, in fact, bear in mind that people are different and have different needs and ways to stay motivated, as well as different values and convictions [20].

This study constituted the first attempt to identify factors that motivate workers and which may lead to increased job productivity in the Greek health care system, providing of course that a measurable outcome related to productivity is recorded in a future study. Within a long-term perspective, this information could help hospital management to increase overall performance, both individual and organizational. The theoretical framework of the study rests on selected elements of well-known motivation theories that have supported similar efforts over the years. Improving motivation usually starts with setting high organizational expectations and by knowing an individual's motivational profile. Maslow and Herzberg both argued that their theories applied to everyone, whereas modern motivation researchers recognize a wide range of individual differences, rather than one universal approach [21].

In this study, the ranked order of motivating factors was i) achievements, ii) remuneration, iii) co-workers and iv) job attributes, although "remuneration" and "co-workers" were practically tied. The same order was replicated within each professional subgroup, thus increasing confidence in the validity of the results. A comparison to Maslow's need-hierarchy theory can provide some interesting insight. The highest ranked motivator, achievements, is a self-actualizing factor covering intrinsic needs such as pride, appreciation, respect and social acceptance. According to Maslow, after meeting previous levels of need, an employee will pursue self-actualization, take risks, learn new things and generally grow in the work environment. The second ranked motivator, remuneration, is a physiological factor which reflects safety needs such as security and stability. These could be met by providing a safe working place, good benefits including retirement, and insurance, which are motivators advancing employee welfare and ensuring that future needs are met.

The other equally valued motivator, co-workers, is related to social needs such as belongingness, relationships and acceptance in formal and informal work groups. This is equivalent to Maslow's third level and a comfortable work environment and open communication can provide these necessities. The lowest ranked factor, job attributes, is associated with accomplishment, creativity, and growth, i.e. *self- esteem *according to Maslow's terminology. When these needs exist, they are among the strongest internal motivators. By providing employees with challenging projects which utilize their talents and help them develop new skills, mangers can help them be successful.

In this study, the range of motivational factors was mixed and Maslow's conclusions that lower level motivational factors must be met before ascending to the next level were not confirmed. The hierarchy of need advocated in Maslow's theory is not immutable and some levels are met in a different order or some are even ignored. A similar outcome has been reported elsewhere [22]. Interestingly, Herzberg's two-factor theory was also not confirmed. Specifically, *remuneration *and *co-workers *are hygiene factors and while these do not motivate, they can satisfy employees if handled properly, but if absent can provoke job dissatisfaction. On the other hand, *job attributes *and *achievement *are motivators, and create satisfaction by fulfilling an individual's higher needs. Their absence does not lead to dissatisfaction, however if met they will, according to Herzberg, promote job satisfaction and encourage production.

Our highest ranked motivator was achievements, implying that work meaningfulness, appreciation and respect were regarded as powerful driving forces and were ranked higher than securing hygiene factors such as salary, social belongingness and work collegiality. This motivator was strongly associated to increasing age, particularly in the physician and nurse subgroups. Being achievement-oriented can be seen as self-actualization. Achieving and the drive to become what one is capable of becoming or to surpass others can be very motivating [23].

Contrarily, remuneration (and salary in particular) was a significant motivator only for professionals in managerial positions. Money is obviously essential for satisfying needs, however it has been suggested that money incentives motivate only if employees perceive a strong linkage between performance and rewards [20,23]. Excessive focus on financial incentives in the public sector could lead to negative consequences. Workers may begin to view financial rewards as more important than other rewards (e.g., praise from supervisors or appreciation by the community), and experience a conflict between their own notion of public sector values and messages about working for financial gain [24]. In the case of the physicians -as the main providers of health care services- this is consistent with the growing literature on the importance of non-monetary work circumstances on physicians' overall work satisfaction [25]. Moreover, most studies on motivation of healthcare workers have identified the need for financial and non-financial incentives as a strategy to increase employee retention [26].

Health professionals in private hospitals were affected by all motivational factors more than their public-hospital counterparts, a result most likely due to the differences in organizational and management styles, which in turn may be due to differences in worker recruitment and selection between the two sectors. Private hospitals obviously aim to maximize their profit, and in light of this, they are at liberty to align their human resource management policies to this goal. This implies the ability to offer both financial and non-financial incentives to physicians, nurses and other professionals who are willing to support the hospital's profit-making goal. The end result is that intrinsic individual needs and external job-related aspects can be concurrently satisfied, leading to satisfied employees who are motivated to perform better. To ascertain that the results regarding the public and private sectors were indeed a result of the organizational differences mentioned, and not due to the confounding effect of demographic variables, ANCOVA was used to control for these potential effects (results not shown but are available upon request). The results were confirmed for all professional categories and all motivational factors.

In Greece, the enforcement of national legislation in 2001, initiated a new era in the healthcare system, in which technical and economic efficiency were set as major targets for improvement. Hospital administrations would be accountable for balancing the effectiveness of provided services, with the efficient use of resources. The spotlight obviously falls on the health care workers in this labor-intense environment. In today's economically unstable environment hospital managers, particularly in the public sector, are restricted in providing additional financial incentives, which could motivate employees to perform better. Therefore, it is important to understand what else might satisfy health professionals in the workplace, and motivate them to improve performance. The next step would be to attempt to measure performance before and after the implementation of a motivation strategy, in order to determine if such incentives are indeed related to improving productivity in Greek (or other countries) hospitals.

The main limitation of this study, which could have implications on the generalizability of the results beyond this group of professionals, has to do with the sample itself. More than three quarters of the respondents worked in public-sector hospitals of the Greek NHS, in which management is limited in its capability of enforcing effective human resource management policies, as was previously mentioned. The employees themselves, being fully aware of this reality, may have "adapted" their responses towards what they regard as feasible, rather than to what would be actually plausible. Further studies, particularly ones using qualitative methods, might shed more light in this area.

## Conclusion

The results of this study are in agreement with the majority of the literature which focuses attention to management approaches employing both monetary and non-monetary incentives to motivate health care workers. Hospital employees report being motivated more by intrinsic factors, implying that these should be a target of effective employee motivation. Furthermore, existing individual differences should be a concern in the manager's motivational agenda, despite this indeed being sometimes unfeasible. The next best thing is strategies for specific demographic and professional subgroups, exploiting empirical information from studies such as the present. The problems and solutions to motivation issues can be complex, and thus research and the timeless theories of Maslow, Herzberg and others (despite not ever having received empirical support from research) can offer ideas and solutions to motivation problems.

## Competing interests

The authors declare that they have no competing interests.

## Authors' contributions

NK was responsible for finalizing the instrument and drafting the manuscript. VP was responsible for developing the initial version of the instrument, conducting the literature review and acquiring, analyzing and interpreting the data. DN was responsible for conception of the study and assisted in interpreting the results and revising the manuscript for intellectual content. All authors have read and approved the final manuscript.

## Pre-publication history

The pre-publication history for this paper can be accessed here:


